# Neither an Optimist Nor a Pessimist Be: Mistaken Expectations Lower Well-Being

**DOI:** 10.1177/0146167220934577

**Published:** 2020-07-06

**Authors:** David de Meza, Chris Dawson

**Affiliations:** 1 London School of Economics and Political Science, UK; 2 University of Bath, UK

**Keywords:** unrealistic optimism, well-being, decision making, loss aversion, disappointment aversion

## Abstract

This article speaks to the classic view that mental health requires accurate self-perception. Using a representative British sample (*N* = 1,601) it finds that, as measured by two established well-being indicators, those with mistaken expectations, whether optimistic or pessimistic, do worse than realists. We index unrealistic optimism as the difference between financial expectations and financial realizations measured annually over 18 years. The effects are not small, with those holding the most pessimistic (optimistic) expectations experiencing a 21.8% (13.5%) reduction in long-run well-being. These findings may result from the decision errors and counteracting emotions associated with holding biased beliefs. For optimists, disappointment may eventually dominate the anticipatory feelings of expecting the best while for pessimists the depressing effect of expecting doom may eventually dominate the elation when the worst is avoided. Also, plans based on inaccurate beliefs are bound to deliver worse outcomes than would rational expectations.

“Pessimism is, in brief, playing the sure game. You cannot lose at it; you may gain. It is the only view of life in which you can never be disappointed. Having reckoned what to do in the worst possible circumstances, when better arise, as they may, life becomes child’s play.”

—Thomas Hardy

“. . . that sanguine expectation of happiness which is happiness itself.”

—Jane Austen, Sense and Sensibility

## Introduction

Is it best to expect the best? Research into dispositional optimism—generalized outcome expectancies that good things will happen (Scheier et al., 1994)—finds that positive beliefs are fundamental for a variety of different positive psychological and physical health-relevant outcomes (Scheier & Carver, 1987, 1992, 1993). Here, positive beliefs are advantageous, as those with an optimistic disposition are thought to be able to cope in a more adaptive way to stressful situations (Scheier & Carver, 1993). However, demonstrating the benefits of dispositional optimism is not straightforward. A finding that positive beliefs are associated with higher well-being may partly reflect the realistic expectation of people likely to have positive experiences. This problem is eliminated by examining how beliefs affect well-being controlling for outcomes. That is, by adopting the alternate psychological perspective of unrealistic optimism. Here, optimism is viewed as a preponderance toward positive forecasting errors or, more formally, as the tendency to overestimate the likelihood of positive events, and underestimate the likelihood of negative events (Weinstein, 1980; Weinstein & Klein, 1996). Unrealistic optimism has been found to be one of the most pervasive human traits, with studies consistently reporting that a large majority of the population (about 80% according to most estimates) display an optimism bias (Sharot, 2011). The bias tends to be highest when events are perceived to be under the individual’s control (Kahneman et al., 1982), that is, when outcomes can be influenced through effort or ingenuity (Harris et al., 2008). However, unrealistic optimism has also been documented for purely chance events (Langer & Roth, 1975). Despite the prevalent tendency for humans to make systematically biased probability assessments, there is still considerable debate within psychology and economics as to whether such beliefs are aligned with psychological well-being.

There is evidence that unrealistic optimism comes with costs as well as benefits. On the downside, when expectations are not fulfilled, a variety of negative emotions are triggered (Diener et al., 1991) including disappointment (Bell, 1985). Even when expectations are fulfilled, there is the loss of the elation that might otherwise be experienced. In general, optimistic beliefs reduce the pleasure from realized outcomes as the emotions derived from outcomes are determined in part by counterfactual thinking—good outcomes are more pleasing when they are unexpected, and negative outcomes feel less disappointing when they are expected (Medvec et al., 1995; Mellers et al., 1997; Shepperd & McNulty, 2002; Sweeny & Shepperd, 2010). For example, Rutledge et al. (2014) find that momentary happiness depends on whether a gamble does better than its objectively expected value. In general, happiness is not determined by how well things are going, but whether they are going better than expected. Among many illustrations of this, McGraw et al. (2004) reported that the most confident basketball players experienced less enjoyment from successful shots and more pain from failed shots.

Loss aversion (Kahneman & Tversky, 1979)—the tendency to feel more pain when experiencing losses than pleasure from equal gains—also implies that optimism has a cost. The utility of an income realization depends on how it compares to a reference level, identified by Kőszegi and Rabin (2006) as expected income. As falling short of the reference has greater cost than the gain of exceeding it, the implication is that the high reference point of optimists makes them worse off than pessimists.

Unrealistic optimism also has benefits. A substantial body of research has documented that positive illusions about the self—unrealistically positive self-evaluations, exaggerated perceptions of control, and unrealistic optimism—are characteristics that bring about and maintain psychological well-being (Alloy & Ahrens, 1987; Pyszczynski et al., 1987; Taylor & Brown, 1988).^1^ The ability to feel good about oneself may be useful when encountering negative feedback or stressful events (Taylor & Brown, 1988). It could also be that human cognition acts in a way more aligned to the philosophy *ignorance is bliss*.

There is also considerable evidence that individuals derive pleasure and pain directly from their beliefs. Loewenstein (1987) argued that most people regularly experience emotions related to anticipation, with the expectation of favorable outcomes having an impact on immediate well-being. Experimental evidence by Berns et al. (2006) found that being told about an impending electric shock was a direct source of misery. Indeed, Lazarus (1966) documented that certain forms of physical pain have no impact on psychological stress over and above that produced by the anticipation of physical pain. That people derive pleasure from positive beliefs and anxiety from negative beliefs, suggests that individuals can be motivated to maintain an optimistic view of themselves and their future (Brunnermeier & Parker, 2005; Sharot & Garrett, 2016).

Misperceptions thus have advantages and disadvantages. Realism also has its advocates. There is a long tradition in Western thought, as inscribed in the temple at Delphi, that to “know thyself” is the right maxim to live by. It is a theme echoed by mid-century humanistic psychology. For instance, Maslow (1950) takes the view that “healthy individuals find it possible to accept themselves and their own nature without chagrin or complaint” (p. 155) and Rogers (1961) “psychotherapy . . . is a process whereby man becomes his organism-without self-deception without distortion” (p. 103). In a review of the dominant views of mental health at the time, Jahoda (1958) concluded that the mentally healthy person was someone “able to take matters one wishes were different, without distorting them to fit these wishes—that is, without inventing cues not actually existing” (p. 51) and that “the perception of reality is called mentally healthy when what the individual sees corresponds to what is actually there” (p. 6). Denial may provide temporary relief from stress and anxiety but in the longer run you have to live in the world as it is not as you would like it to be. Failure to recognize this will lead to stressful dissonance and poor decision making. Denial is at most a first step in a healthy response to a shock, such as personal loss, a process that must eventually end with acceptance (Kübler-Ross, 1969).

For mainstream economists, contact with reality and unbiased assessments of probabilities are traditionally viewed as being beneficial. According to this perspective, decisions based on accurate, objective, and unbiased evidence must maximize expected utility.^2^ Unrealistic optimism, like any judgmental bias, distorts the decision making process, leading to sub-optimal outcomes and lower well-being. Faulty assessments do not only result in systematic decision errors, but also lead to rash behavior (de Meza et al., 2019) and inadequate precautionary measures (Dillard et al., 2009).

The long-term consequences of optimism on well-being are therefore ambiguous. Depending upon the intensity of anticipatory emotions, loss aversion and the costs of distorted decision making, optimistic, pessimistic, or even realistic beliefs could emerge as utility maximizing. In fact, as we have shown above, the evolving paradigms in psychology and economics have at some point argued in favor of each type of belief as being fundamental to contentment. More than this, whether optimism is beneficial, harmful, or neutral may depend on its level. For instance, while a little bit of optimism may be beneficial, extreme standings on potentially “desirable” personality traits might be maladaptive (Carter et al., 2018). Thus, for optimism, more may not always be better—the benefits of extra optimism may be diminishing and the costs increasing.

As it is not possible to sign the effect of biased beliefs on well-being from theoretical considerations alone, there is a need for evidence. This article uses a large and lengthy longitudinal survey to establish whether, taking everything into account, it is unrealistic optimists, unrealistic pessimists, or realists that have the highest long-run well-being. It is quite rare to be able to measure forecasting errors on a repeated basis. This we can do, but only in one domain, household finances. Whether being unrealistically optimistic about financial prospects is correlated with unrealistic optimism in other non-financial domains remains an open question. Although we have some arguments that unrealistic optimism is likely to be domain-general, all we can rigorously claim is that realism over financial forecasts is associated with higher well-being, measured by life satisfaction and psychological distress, than is unrealistic optimism or pessimism.

## Method

### Participants

The data used for analysis are the British Household Panel Survey (BHPS) 1991 to 2009 (Waves 1–18). The BHPS is a nationally representative longitudinal survey of more than 5,000 households (comprising approximately 12,000 individuals) which began in 1991, funded by the UK Economic and Social Research Council as a national and international multi-purpose research resource. The questionnaire instrument includes a household questionnaire and a lengthy individual questionnaire covering a range of topics including household composition, demographic characteristics, economic activity, health, and finances. The sample used for the subsequent analysis is restricted to the original BHPS sample covering Great Britain and to individuals who were observed in all 18 waves and gave valid responses to the dependent and independent variables used in the subsequent analysis. This yields a balanced panel of 1,601 individuals.

### Measures

#### Optimism

By optimism, we do not mean a belief that good things will happen. Such a belief may be justified, making it impossible to distinguish biased from realistic expectations. It is unrealistic optimism that we are concerned with. That is, an excessive belief in the probability of good realizations and therefore a preponderance of positive forecast errors. Various methodological approaches have been used to assess the extent of unrealistic optimism in the population. One major problem faced by these studies is the difficulty in determining the objective probabilities against which expectations should be compared. In many cases the average probability (risk) of the population is used as an objective probability, which can lead to individuals frequently being misclassified as unrealistically optimistic. For instance, a man who assesses his risk of bowel cancer at 2% could be classed as optimistic, when we consider the risk for men in general is much higher. However, this may not imply bias. Some people are objectively less (more) likely to experience bad (good) events. Hence, for optimism to be unrealistic, predictions need to be compared with later experiences or statistically derived true expected values (Coelho, 2010; Weinstein & Klein, 1996). This is the approach we adopt in our empirical analysis, by utilizing two questions asked in every wave of the BHPS. First, the BHPS asks individuals “Looking ahead, how do you think you yourself will be financially a year from now; better than you are now, worse than you are now, or about the same?” It also asks “Would you say that you yourself are better off, worse off or about the same financially than you were a year ago?”^3^ Comparing financial expectations at timet (of t+1) with the financial realization at timet+1 over the 18 waves provides the basis for measuring optimism. What subjects understand by being “better off” or “worse off” financially is not straightforward. A further question asks subjects to attribute the main reason for why their financial situation changed. For those who were financially “better off,” 57% report that an income increase is the main reason, followed by 15% who report a fall in expenditures. For those who were financially “worse off,” 50% report that the reason is higher expenditures, whereas 28% report lower income. In judging unrealistic optimism, it is not obviously the source of the change in finances which is relevant. What matters is that individual who reported a financial change, objectively experienced that change. Using the same data source as us, Brown and Taylor (2006) check how consistent intertemporal judgments of change in financial situation are with actual changes in real and nominal income, with the latter calculated from the difference in reports of income level 1 year to the next (i.e., timet−1 to t). The results reassuringly report consistency between the two measures, suggesting that individual perceptions square with what happens to actual finances.^4^

Responses to the financial expectation and realization questions are both assessed on a 3-point scale (from −1 to +1) ranging from “worse off” to “better off.” Measured discrepancies between expectations and realizations in a particular time period can be decomposed into a permanent component reflecting a systematic psychological bias—a stable individual trait associated with generally biased expectations—and a transitory component reflecting random shocks to realizations and random errors of evaluation. As it is the effects of the psychological component that we are concerned with, transitory optimism is minimized by constructing for each individual (i), a time-averaged expectation $\left({\bar{E}}_{i}\right)$, and realization variable $\left({\bar{R}}_{i}\right)$ over the available data (for a scatterplot of ${\bar{E}}_{i}$ and ${\bar{R}}_{i}$, see Figure S1 in the Supplemental Material). From these variables we can also construct a scale (from −2 to +2) of financial forecast error, ${\bar{FE}}_{i}={\bar{E}}_{i}-{\bar{R}}_{i}$. Here, positive forecast errors (i.e., scores above zero) are associated with optimistic beliefs and negative forecast errors with pessimistic beliefs.

#### Well-being

Psychological well-being is captured by responses to the 12-item General Health Questionnaire (GHQ) and to a question on life satisfaction (LS). The GHQ is carried out in every wave of the BHPS and is a widely applied measure and arguably one of the most reliable indicators of psychological distress or “disutility” (Argyle, 1989). Moreover, the GHQ in the BHPS has been shown to be robust to retest effects making it highly suitable for longitudinal analysis (Pevalin, 2000). We use the Likert-type scoring method, where responses to the 12 items are coded on a 4-point (items scored 0-1-2-3) scoring system that ranges from a “disagree strongly” to “agree strongly.” Scores are then summed together, providing a total GHQ score ranging from 0 to 36, with higher scores corresponding to lower psychological well-being or higher “disutility.” An alternative scoring method is the “Caseness” scoring method, which sums the number of times the respondent places themselves in the fairly stressed or highly stressed category (items scored 0-0-1-1), providing a total GHQ score ranging from 0 to 12. However, Banks et al. (1980) suggest that the Likert-type method is to be preferred to the “Caseness” method in studies using parametric multivariate techniques, since its distribution more closely approximates the normal. Nevertheless, all the results presented in the subsequent analysis are robust to the “Caseness” method.

Responses to the LS question are given on a 7-point Likert-type scale, where respondents were asked in waves 6 to 10 and 12 to 18 “How dissatisfied or satisfied are you with your life overall?” ranging from “not satisfied at all” to “completely satisfied.” LS is commonly used by psychologists and sociologists as a measure of an individual’s psychological state and has been shown to be strongly correlated with other survey instruments designed to capture subjective well-being (Diener et al., 1999). For each individual i, we construct a time-averaged measure of well-being ${\bar{W}}_{i}^{j}$, for well-being measure j=(GHQ,LS) over the available data (for the full distribution of these two well-being variables, see Figure S2 in the Supplemental Material).

#### Demographic characteristics

A number of other variables may explain the correlation between well-being and financial expectations and realizations. As covariates we include a control for gender and a range of individual time-averaged socioeconomic and sociodemographic controls. These control variables are age (in linear form); marital status; the number of dependent children in the household; economic activity; educational attainment; housing tenure; logged monthly household income (deflated); and number of cigarettes smoked and region of residence. All these controls have been shown to be strong predictors of subjective well-being (Dolan et al., 2008; Kahneman & Krueger, 2006).

Table S1 in the Supplemental Material presents summary statistics for our time-averaged demographic characteristics and our time-averaged measures of expectations, realizations, forecast errors, and psychological well-being. Consistent with much of the literature, financial expectations exceed realizations, therefore our sample is on average optimistic. This is confirmed by our financial forecast error variable, where the mean exceeds zero. Mean GHQ is 10.90 and mean LS is 5.33. The mean age is approximately 48 years. Just less than 55% of the sample is female and 13% report holding a university or college degree.

### Analytic Strategy

As we have argued, expectations have multiple opposing effects on well-being and these effects may not be linear. This raises the possibility that the relation between expectations and well-being is not monotonic. We therefore need a functional form that is sufficiently flexible to capture this property. Specifically, we need to (a) allow expectations and realizations to separately influence well-being, (b) allow relationships to be potentially nonlinear, and (c) allow for the effect of expectations on well-being to depend on the level of realization. A flexible form that can handle all these possibilities and is easily estimated is the general second-degree polynomial. Using between-person variation, we estimate the following equation using ordinary least squares (to simplify, we omitted all control variables from the notation):

(1) $${\bar{W}}_{i}^{j}=b_{0}+b_{1}{\bar{E}}_{i}+b_{2}{\bar{R}}_{i}+b_{3}{\bar{E}}_{i}^{2}+b_{4}{\bar{R}}_{i}^{2}+b_{5}\left({\bar{E}}_{i}\times {\bar{R}}_{i}\right)+e_{i},$$

where ${\bar{W}}_{i}^{j}$ is our time-averaged measure of well-being for individual i, and well-being measure j=(GHQ,LS). Our five individual time-averaged polynomial terms are as follows: $b_{1}{\bar{E}}_{i}$ (expectations), $b_{2}{\bar{R}}_{i}$ (realizations), $b_{3}{\bar{E}}_{i}^{2}$ (expectations × expectations), $b_{4}{\bar{R}}_{i}^{2}$ (realizations × realizations), and $b_{5}({\bar{E}}_{i}\times \;{\bar{R}}_{i})$ (expectations × realizations). Here,$b_{3}$ and $b_{4}$ capture any nonlinear effects of expectations and realizations on well-being, respectively. If the effect of expectations on well-being differ by the level of realizations this is measured by $b_{5}$.

Equation 1 embodies a between-person approach. Instead, a within-person estimation could be undertaken. Here, the well-being of individual i at time t is dependent on an individual fixed effect $a_{i}$, and on $R_{it},\;E_{it},\;E_{it-1}$. The problem is that if optimism is a stable individual characteristic, the effect of optimism on well-being cannot be captured by a within-person approach any more than could the effect of say, gender on well-being. To the extent to which optimism is a stable trait, the within-person equation only measures the effect of random fluctuations in realizations and changes in expectations caused by either random errors of evaluation or updating in light of new circumstances. It does not provide a way to draw a conclusion about how within-person changes in optimism, if they occur, affect well-being.^5^

## Results

We begin by examining the results from our second-degree polynomial equation with the full set of control variables included. Then we examine the results from a special case of our second-degree polynomial equation, which includes the forecast error variable directly. Finally, the robustness of our results is investigated by estimating the equations with all possible combinations of our control variables. Finally, we investigate whether biased beliefs may be correlated with some personality feature that is incompatible with well-being.

It is worth reiterating before we discuss our results that lower GHQ scores represent higher well-being, whereas the opposite is true for our LS score. Table 1 displays the results of the second-degree polynomial analysis. Here, we see that the three second-degree polynomial terms—that is, ${\bar{E}}_{i}^{2},\;{\bar{R}}_{i}^{2},\;\mathrm{and}{\bar{E}}_{i}\times \;{\bar{R}}_{i}$—were jointly significant in predicting GHQ, *F*(3, 1565) = 5.02, *p* < .001, and LS, *F*(3, 1565) = 8.75, *p* < .001. Regression 1 of Table 1 displays that the quadratic relationship between expectations and GHQ is highly statistically significant (*b* = 2.66, 95% CI = [1.12, 4.20], *t* = 3.39, *p* = .001). Regression 2 of Table 1 displays that this is also the case for LS (*b* = −0.95, 95% CI = [–1.36, –0.54], *t* = −4.56, *p* < .001). Moreover, in both Regressions 1 and 2 of Table 1, the effect of expectations on well-being differs by the level of realizations: (Regression 1: *b* = −4.18, 95% CI = [–6.46, –1.91], *t* = −3.60, *p* < .001; Regression 2: b=0.86,95%CI=[0.26,1.47],t=2.79,p=.005). These results together suggest that our second-degree polynomial fits the data well and importantly, that the relationship between expectations and well-being is nonlinear and depends upon the level of the realization.

**Table 1.** Second-Degree Polynomial Ordinary Least Squares Regressions Measuring the Impact of Expectations and Realizations on Psychological Well-Being.

Predictors	Dependent variable: GHQ				Dependent variable: Life satisfaction			
	Regression 1				Regression 2			
	b	95% CI		t(*df* = 1,565)	b	95% CI		t(*df* = 1,565)
		Low	High			Low	High	
${\bar{E}}_{i}$	−1.35	−2.22	−0.48	−3.05**	0.22	−0.01	0.45	1.84
${\bar{E}}_{i}^{2}$	2.66	1.12	4.20	3.39**	−0.95	−1.36	−0.54	−4.56**
${\bar{R}}_{i}$	−0.88	−1.61	−0.15	−2.38*	0.44	0.25	0.64	4.47**
${\bar{R}}_{i}^{2}$	1.60	0.04	3.16	2.01*	−0.42	−0.84	−0.01	−1.99*
${\bar{E}}_{i}\times {\bar{R}}_{i}$	−4.18	−6.46	−1.91	−3.60**	0.86	0.26	1.47	2.79**
*F*-test	5.02**				8.75**			
*R* ^2^	.173				.195			
N	1,601				1,601			

*Note.* All regressions include a control variable for gender and a series of individual time-averaged control variables for age (in linear form), marital status, the number of dependent children in the household, economic activity, educational attainment, housing tenure, logged monthly household income (deflated), number of cigarettes smoked and region of residence. Full results are available on request. *F*-test is for the three second-degree polynomial terms. CI = confidence interval; GHQ = General Health Questionnaire.

* *p* < .05. **p < .01.

**Figure 1.**
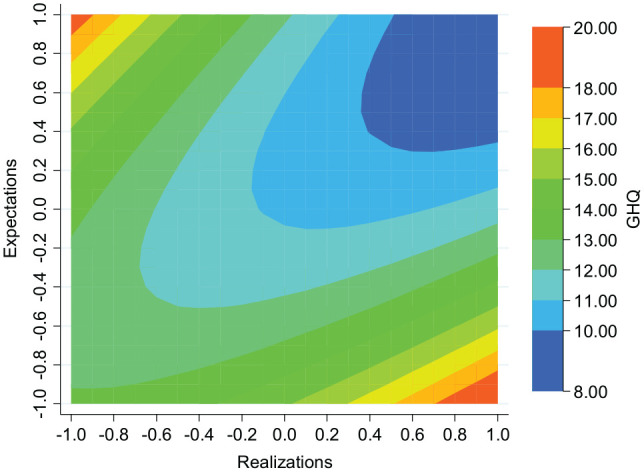
Contour map illustrating the predicted relationship between GHQ, expectations, and realizations (Regression 1, Table 1). We use the thin-plate-spline interpolation method.

**Figure 2.**
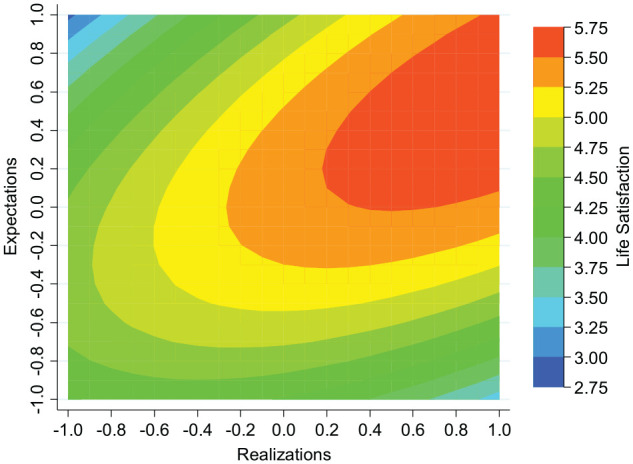
Contour map illustrating the predicted relationship between life satisfaction, expectations, and realizations (Regression 2, Table 1). We use the thin-plate-spline interpolation method.

The key results of the second-degree polynomial equation are summarized in the contour maps presented in Figures 1 and 2, which are useful for graphically illustrating complex nonlinear functions. In each of the figures, the *y*-axis and *x*-axis contain the predictor variables, expectations $\left({\bar{E}}_{i}\right)$ and realizations $\left({\bar{R}}_{i}\right)$, respectively. The *z*-axis in each case represents colored contour bands, representing ranges of the predicted levels of the response variable, psychological well-being $\left({\bar{W}}_{i}^{j}\right)$. In each figure, a 45-degree diagonal line, originating from the origin $\left({\bar{E}}_{i}={\bar{R}}_{i}\right)$ would represent those with realistic beliefs, while the top left and bottom right of each diagram represents those with the most optimistic beliefs and pessimistic beliefs, respectively. In both the GHQ and LS contour maps, given realizations, peak well-being tends to be located around the imaginary 45-degree line, with well-being falling away as expectations vary in optimistic and pessimistic directions. It seems reasonable that an equiproportional increase in realizations and expectations should boost well-being. Reflecting this, psychological well-being increases as we move from realistic beliefs associated with low levels of expectations and realizations to realistic beliefs associated with high levels of expectations and realizations.

In summary, the main results are similar whether well-being is measured by GHQ or by LS. Given realizations, those holding realistic beliefs are best off.

**Figure 3.**
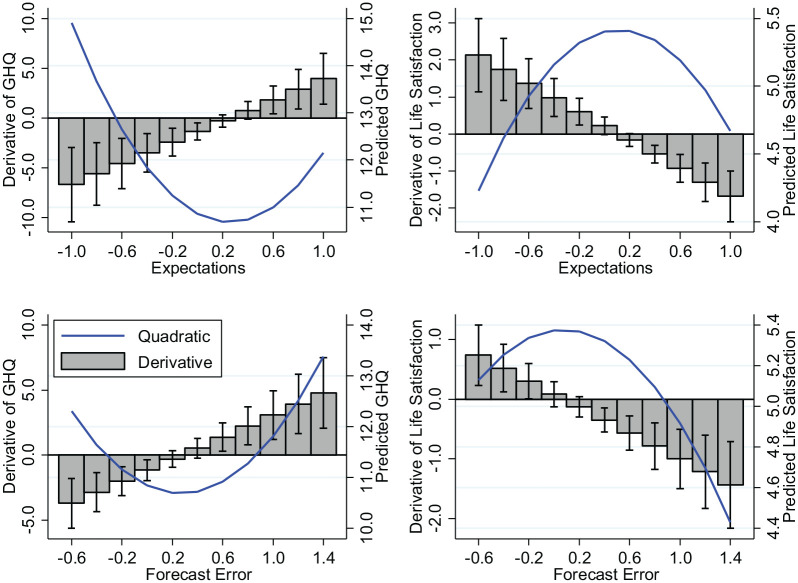
The quadratic relationship between expectations, forecast errors, and psychological well-being. We also include as bar charts the slope estimates (derivative) of the quadratic relationships, with the 95% confidence intervals (CIs) included. Top left (Regression 1, Table 1): the turning point of the quadratic prediction is 0.25, with a 95% CI = [0.13, 0.38]. Top right (Regression 2, Table 1): the turning point of the quadratic prediction is 0.11, with a 95% CI = [0.02, 0.21]. Bottom left (Regression 1, Table 2): the turning point of the quadratic prediction is 0.27, with a 95% CI = [0.12, 0.43]. Bottom right (Regression 2, Table 2): the turning point of the quadratic prediction is 0.08, with a 95% CI = [−0.09, 0.25]. All of these turning points and the respective 95% CI lie comfortably within the range of observable values in the data.

An alternative approach to displaying the results from our second-degree polynomial equations is to plot how expectations affect GHQ and LS for a given level of realization. In the top panels of Figure 3, we display the estimated quadratic relationship between expectations and psychological well-being, evaluated at a realization of zero. Therefore, if realistic beliefs are optimal for well-being, we expect a turning point to emerge where expectations approach zero. The top panels of Figure 3 confirm that this turning point emerges for both the GHQ and LS measures of well-being. The 95% CI for the turning points are well within the observed range of expectations, so we can be confident that reasonably realistic beliefs are associated with the highest well-being. Within the top panels of Figure 3, we also include as bar charts the slope estimates (derivative) of the quadratic relationship between expectations and psychological well-being, with the 95% CI included. These estimates illustrate that the positive and negative slopes of the quadratic relationship are both significantly different from zero. The effects of misperceptions are not small. Specifically, those with the most pessimistic (optimistic) expectations are associated with a 37.2% (11.8%) higher level of GHQ, than those with the most realistic beliefs. The equivalent effects for LS are that those with the most pessimistic (optimistic) expectations experience a 21.8% (13.5%) reduction in well-being. Conclusions are similar when the effect of expectations on psychological well-being is evaluated at other values of the realization.

Next, we examine the effects of mistaken expectations on well-being by means of a special case of our second-degree polynomial equation. Specifically, we conduct a multiple regression in which forecasting errors $\left({\bar{FE}}_{i}={\bar{E}}_{i}-{\bar{R}}_{i}\right)$ and realizations $\left({\bar{R}}_{i}\right)$ were entered as predictors of psychological well-being. To determine whether realistic beliefs are optimal for well-being, forecasting errors is entered in a quadratic form. In other words, we estimated the following equation using ordinary least squares (to simplify, we again omit all control variables from the notation):

(2) $${\bar{W}}_{i}^{j}=c_{0}+c_{1}{\bar{FE}}_{i}+c_{2}{\bar{FE}}_{i}^{2}+c_{3}{\bar{R}}_{i}+e_{i}.$$

Although this is a restricted form of our second-degree polynomial equation, it is a natural formulation and easily interpretable.^6^ Table 2 displays the results. Regression 1 of Table 2 shows that the quadratic relationship between forecasting errors and GHQ is highly statistically significant (b=2.12,95%CI=[1.01,3.23],t=3.75,p<.001). Regres-sion 2 of Table 2 displays that this is also the case for LS (b=−0.54,95%CI=[−0.84,−0.25],t=−3.59,p<.001). In the bottom panels of Figure 3, we display the predicted quadratic relationship between forecasting errors and psychological well-being. If realistic beliefs are optimal for well-being, we expect a turning point to emerge where the forecast error approaches zero. The bottom panels of Figure 3 confirm that this turning point emerges for both the GHQ and LS measures of well-being with 95% CI for the turning points indicating that we can again be confident in concluding that reasonably realistic beliefs are associated with the highest well-being. Within the bottom panels of Figure 3, we also include as bar charts the slope estimates (derivative) of the quadratic relationship between forecasting errors and psychological well-being, with the 95% CI included. These estimates illustrate that the positive and negative slopes of the quadratic relationship are both significantly different from zero. Again, these effects are not small. A one-point increase in forecast error from those with realistic beliefs, increases GHQ by 8.9% and reduces LS by 8.5%. The equivalent comparison when moving in the pessimistic direction generates an increase in GHQ of 30.2% and a reduction in LS of 11.7%.

**Table 2.** Ordinary Least Squares Regressions Measuring the Impact of Forecasting Errors on Psychological Well-Being.

Predictors	Dependent variable: GHQ				Dependent variable: Life satisfaction			
	Regression 1				Regression 2			
	b	95% CI		t(*df* = 1,567)	b	95% CI		t(*df* = 1,567)
		Low	High			Low	High	
${\bar{FE}}_{i}$	−1.16	−1.94	−0.37	−2.90**	0.09	−0.12	0.29	0.79
${\bar{FE}}_{i}^{2}$	2.12	1.01	3.23	3.75**	−0.54	−0.84	−0.25	−3.59**
${\bar{R}}_{i}$	−2.07	−2.68	−1.46	−6.66**	0.54	0.38	0.70	6.49**
*R* ^2^	.172				.188			
N	1,601				1,601			

*Note*. All regressions include a control variable for gender and a series of individual time-averaged control variables for age (in linear form), marital status, the number of dependent children in the household, economic activity, educational attainment, housing tenure, logged monthly household income (deflated), number of cigarettes smoked, and region of residence. Full results are available on request. CI = confidence interval; GHQ = General Health Questionnaire.

* *p* < .05. **p < .01.

Next, we investigate the robustness of our coeffcient estimates by conducting our analysis across all possible combinations of our control variables (Young & Holsteen, 2017). Tables S2 and S3 in the Supplemental Material reports regressions with the same specifications as in Tables 1 and 2, but where the reporting of statistics reflects the mean estimates from the 1,024 unique combinations of control variables. These tests illustrate that our results are strongly robust to model specification and, as such, not dependent on knife-edge specifications. Finally, we investigate an interesting possibility, which is that extreme beliefs may be correlated with some personality feature that is incompatible with well-being. In Wave 15 of our data, we have available the short 15-item Big-Five inventory (BFI-15). Tables S4 and S5 in the Supplemental Material report regressions with the same specifications as in Tables 1 and 2, but adding controls for conscientiousness, extraversion, agreeableness, openness, and neuroticism. In summary, consistent with previous studies personality type is important for well-being (Hayes & Joseph, 2003), this however does not explain the relationship between our well-being measures and optimism. Reasonably realistic beliefs are still associated with the highest well-being (for a graphical representation of these results, see Figure S3 in the Supplemental Material)

## Discussion

This article finds that long-run well-being is higher for realists—those who exhibit long-run accuracy in forecasting their financial outcomes—than for either optimists or pessimists. These effects are not small. Compared with realists, those with the most pessimistic (optimistic) expectations have a 37.2% (11.8%) higher level of psychological distress. For LS, those holding the most pessimistic (optimistic) expectations are associated with a 21.8% (13.5%) reduction in well-being when compared with realists.

Whether the findings are due to counteracting emotions or decision errors is an open question. It could be that as optimism increases, disappointment eventually dominates the anticipatory feelings of expecting the best, so well-being starts to fall. For pessimists, the depressing effect of expecting doom (dread) may eventually dominate the elation when the worst is avoided. Also, plans based on inaccurate beliefs are bound to deliver worse outcomes than would rational expectations. At all events, our finding is that misperception of either sign is bad for well-being.

Unrealistic optimism in the financial domain is a very specific instance of optimism, which raises the question whether our results would be different if we had data on unrealistic optimism in other domains. As unrealistic optimism tends to be greatest when outcomes are perceived to be under the individual’s control and can be influenced through effort or ingenuity, household finances seems likely to elicit high levels of unrealistic optimism. If optimism is domain-general, we would expect our financial measure to be correlated with unrealistic optimism in other settings in which the environment is fertile for eliciting bias in beliefs. To the extent this is the case, our procedure captures the well-being consequences of unrealistic optimism more generally. We are not aware of comparable data for unrealistic optimism over other activities, so we cannot conclusively distinguish whether our results arise because unrealistic optimism is domain-general, or that financial optimism contributes importantly to well-being but is unrelated with optimism in other contexts.

Some evidence that our specific measure of financial optimism may capture a more domain-general bias is provided by de Meza et al. (2019). Using the same data and optimism measure as here, financial optimism is found to be highly correlated with activities which are not directly financial but are plausibly influenced by optimism. In particular, financial optimists are more likely to smoke. The psychology here is that optimists tend to underestimate the occurrence of negative events, such as illness and injury, leading to excessive participation in risky activities such as smoking while undertaking insufficient precautionary interventions.

Despite the inclusion of many controls including personality factors, finding that holders of false beliefs have lower well-being does not ensure the relationship is causal. As we are concerned with the long-run comprehensive effects of an underlying and potentially unchanging misperception propensity, it is difficult to see that there is an alternative to an observational methodology. If there is a causal relationship, there is the issue of the direction of causality. One possibility is that feeling good makes people more positive about the future. The implication is that well-being is monotonically increasing in unrealistic optimism, which we do not find. A second version of reverse causality is that fewer stresses make for more accurate forecasts. We investigated whether within-person changes to well-being increase the accuracy of expectations but find no evidence of this (see Figure S4 in the Supplemental Material).

A further consideration is that optimism might be a (partially) self-fulfilling prophecy. If so, optimists have higher financial realizations than if their expectations were more realistic. Once this is taken into account, even if optimists suffer more disappointment, their well-being could be higher. As our procedure controls for realizations, only the disappointment is captured. It could therefore be falsely concluded that optimism depresses well-being. Excluding realizations as a control variable is not appropriate as well-being is directly affected by financial realizations which, as rational expectations implies, are positively correlated with expectations. If unrealistic optimism does boost financial realizations, the only definite conclusion is that pessimism is worse for well-being than is realism. There is, however, reason to doubt that optimism affects performance. In experiments, Tenney et al. (2015) *inter alia* manipulate optimism but find no significant effect on performance.

There is also the important question of the possibility of bias in the components of our optimism measure. For instance, when asked to judge a change in current financial status relative to a year ago, people may suffer recall bias. There is no problem if positive and negative events are recalled equally, but if negative events are more likely to deteriorate in the memory, financial improvements will be underestimated. The implication is that the level of expectations that maximize well-being occurs at a more pessimistic level than we report. Temporal self-appraisal theory (Wilson & Ross, 2001) has the opposite implication. Here, people may exaggerate any actual improvement by recalling the past as worse than it was, thereby enhancing their present selves by criticizing their past selves (Wilson & Ross, 2000, 2001). If so, financial improvements will be overestimated, implying in our context that the expectations that maximize well-being are more optimistic than we conclude. Despite the possibility of bias in intertemporal comparisons the evidence suggests this is not very important in practice. According to Newby-Clark and Ross (2003) and Seidlitz and Diener (1993) there is little difference in people’s ability to recall both negative and positive events. More directly, in our data there are a strong match between individual’s intertemporal judgments of change in financial situation and the changes constructed from annually reported levels of household income (Brown & Taylor, 2006). This indicates recall bias of either type is not a problem. Finally, people may also show a bias in forecasting their future financial situation. However, this of course is the focus of our article.

A number of papers on counterfactual comparisons experimentally examine one aspect of mistaken beliefs, the tendency of optimists to experience disappointment when their expectations are dashed (Medvec et al., 1995; Mellers et al., 1997; Shepperd & McNulty, 2002). Sweeny and Shepperd (2010) also study disappointment but include a potential benefit of optimism, reduction of anticipatory negative affect. In their study, students are asked to forecast their exam result immediately before it is revealed. Affect is measured at time of forecast and when the result is disclosed. Here, the affective costs of positive expectations were found to outweigh the benefits, suggesting it is better to be pessimistic. However, measuring affect immediately before announcing results gives little opportunity to savor success. Measured a month earlier, optimists may be more relaxed than pessimists. Nonetheless, how do we reconcile these results with our finding that well-being is lower for both pessimists and optimists alike?

In addressing this, it is important to recognize that unlike the studies mentioned above, our study is not specific to the momentary emotions associated with optimism, whether that be the anticipatory utility from positive expectations or the disappointment when outcomes fall short of expectations. That anticipatory utility exceeds disappointment (or the opposite) is not sufficient to conclude that optimism leads to greater (lower) well-being. For instance, well-being may depend on aspects of optimism not captured by its impact on emotions. Greater optimism may be associated with more accidents or inappropriate savings decisions. In the same way, pessimists may forego worthwhile opportunities or take excessive precautions. Indeed, Sweeny and Shepperd (2010) acknowledge that there may be other costs of pessimism not captured in their experiment. More than this, the well-being consequences of decision errors may in certain domains be momentary but in others take years to materialize. In a similar way, a current episode of disappointment may quickly evaporate or alternatively lead to serious longer-term problems such as chronic stress and depression (Brown & Harris, 2001). Our procedure takes all of these additional aspects of optimism into account, so answers the question of whether optimism is associated with higher well-being. Results can therefore differ considerably from studies that focus on the well-being consequences of a singular aspect of optimistic thinking.

Although unrealistic optimism prevails in the population, according to our results, it is not a recipe for maximizing well-being. This might seem to pose an evolutionary puzzle, except that reproductive success not well-being is the “mission” of evolution.

## Supplemental Material

PSPB_Methods_File – Supplemental material for Neither an Optimist Nor a Pessimist Be: Mistaken Expectations Lower Well-Being Click here for additional data file. Supplemental material, PSPB_Methods_File for Neither an Optimist Nor a Pessimist Be: Mistaken Expectations Lower Well-Being by David de Meza and Chris Dawson in Personality and Social Psychology Bulletin

Supplemental_Materials_v1.1 – Supplemental material for Neither an Optimist Nor a Pessimist Be: Mistaken Expectations Lower Well-Being Click here for additional data file. Supplemental material, Supplemental_Materials_v1.1 for Neither an Optimist Nor a Pessimist Be: Mistaken Expectations Lower Well-Being by David de Meza and Chris Dawson in Personality and Social Psychology Bulletin

## Notes

1. Many of these studies are based on the misconception that people who report that they are positive about their future are demonstrating a bias (see Colvin & Block, 1994; Weinstein & Klein, 1996). Some people are objectively more likely to experience good events and less likely to experience bad events. For example, Dunning and Story (1991) found that although depressed people have lower expectations then the non-depressed, their outcomes were even worse, so depressives emerged as more unrealistically optimistic than non-depressives. For optimism to be unrealistic, predictions need to be compared with later experiences or statistically derived true expected values (Coelho, 2010; Weinstein & Klein, 1996) as is done in our empirical analysis.

2. The exception is if false beliefs influence others, as in Trivers (2000).

3. The alternative to the 3-point scale would be something like, “Please indicate your forecast of income change, where 0 = *no change*, 3 = *highest positive change imaginable*, and −3 = *highest negative change imaginable*.” This is problematic because whereas increase has an objective meaning, Magnitude 2 is quite subjective.

4. If there is misreporting but it is random this will not bias our coefficients. Even most versions of systematic bias would not affect the conclusion that there is a turning point of well-being with respect to beliefs.

5. It is possible that an individual’s optimism may change in that the extent to which they systematically overestimate outcomes alters. There is no variable that directly captures this effect as a change in optimism in a given year may just reflect random variation in realizations or expectations.

6. Equation 2 is obtained from Equation 1 by setting $b_{1}=-b_{2}$ and $b_{3}=-b_{4}=2b_{5}$, restrictions which are not satisfied in the estimate of Equation 1.

## References

[bibr1-0146167220934577] Alloy L. B. Ahrens A. H. (1987). Depression and pessimism for the future: Biased use of statistically relevant information in predictions for self versus others. Journal of Personality and Social Psychology, 52, 366–378. 3559896 10.1037//0022-3514.52.2.366

[bibr2-0146167220934577] Argyle M. (1989). The psychology of happiness. Routledge.

[bibr3-0146167220934577] Banks M. H. Clegg C. W. Jackson P. R. Kemp N. J. Stafford E. M. Wall T. D. (1980). The use of the General Health Questionnaire as an indicator of mental health in occupational studies. Journal of Occupational Psychology, 53, 187–194.

[bibr4-0146167220934577] Bell D. E. (1985). Disappointment in decision making under uncertainty. Operations Research, 33, 1–27.

[bibr5-0146167220934577] Berns G. S. Chappelow J. Cekic M. Zink C. F. Pagnoni G. Martin-Skurski M. E. (2006). Neurobiological substrates of dread. Science, 312, 754–758. 16675703 10.1126/science.1123721 PMC1820741

[bibr6-0146167220934577] Brown G. W. Harris T. O. (2001). Social origins of depression: A study of psychiatric disorder in women. Routledge.

[bibr7-0146167220934577] Brown S. Taylor K. (2006). Financial expectations, consumption and saving: A microeconomic analysis. Fiscal Studies, 27, 313–338.

[bibr8-0146167220934577] Brunnermeier M. K. Parker J. A. (2005). Optimal expectations. The American Economic Review, 95, 1092–1118.

[bibr9-0146167220934577] Carter N. T. Miller J. D. Widiger T. A. (2018). Extreme personalities at work and in life. Current Directions in Psychological Science, 27, 429–436.

[bibr10-0146167220934577] Coelho M. P. (2010). Unrealistic optimism: Still a neglected trait. Journal of Business and Psychology, 25, 397–408.

[bibr11-0146167220934577] Colvin C. R. Block J. (1994). Do positive illusions foster mental health? An examination of the Taylor and Brown formulation. Psychological Bulletin, 116, 3–20. 8078973 10.1037/0033-2909.116.1.3

[bibr12-0146167220934577] de Meza D. Dawson C. Henley A. Arabsheibani G. R. (2019). Curb your enthusiasm: Optimistic entrepreneurs earn less. European Economic Review, 111, 53–69.

[bibr13-0146167220934577] Diener E. Colvin C. R. Pavot W. G. Allman A. (1991). The psychic costs of intense positive affect. Journal of Personality and Social Psychology, 61, 492–503. 1941521

[bibr14-0146167220934577] Diener E. Suh E. M. Lucas R. E. Smith H. L. (1999). Subjective well-being: Three decades of progress. Psychological Bulletin, 125, 276–302.

[bibr15-0146167220934577] Dillard A. J. Midboe A. M. Klein W. M. (2009). The dark side of optimism: Unrealistic optimism about problems with alcohol predicts subsequent negative event experiences. Personality and Social Psychology Bulletin, 35, 1540–1550. 19721102 10.1177/0146167209343124

[bibr16-0146167220934577] Dolan P. Peasgood T. White M. (2008). Do we really know what makes us happy? A review of the economic literature on the factors associated with subjective well-being. Journal of Economic Psychology, 29, 94–122.

[bibr17-0146167220934577] Dunning D. Story A. L. (1991). Depression, realism, and the overconfidence effect: Are the sadder wiser when predicting future actions and events? Journal of Personality and Social Psychology, 61, 521–532. 1960645 10.1037//0022-3514.61.4.521

[bibr18-0146167220934577] Harris P. R. Griffin D. W. Murray S. (2008). Testing the limits of optimistic bias: Event and person moderators in a multilevel framework. Journal of Personality and Social Psychology, 95, 1225–1237. 18954204 10.1037/a0013315

[bibr19-0146167220934577] Hayes N. Joseph S. (2003). Big 5 correlates of three measures of subjective well-being. Personality and Individual Differences, 34, 723–727.

[bibr20-0146167220934577] Jahoda M. (1958). Current concepts of positive mental health. Basic Books.

[bibr21-0146167220934577] Kahneman D. Krueger A. B. (2006). Developments in the measurement of subjective well-being. Journal of Economic Perspectives, 20, 3–24.

[bibr22-0146167220934577] Kahneman D. Slovic P. Tversky A. (1982). Judgment under uncertainty: Heuristics and biases. Cambridge University Press. 10.1126/science.185.4157.1124 17835457

[bibr23-0146167220934577] Kahneman D. Tversky A. (1979). Prospect theory: An analysis of decision under risk. Econometrica: Journal of the Econometric Society, 47, 263–291.

[bibr24-0146167220934577] Kőszegi B. Rabin M. (2006). A model of reference-dependent preferences. The Quarterly Journal of Economics, 121, 1133–1165.

[bibr25-0146167220934577] Kübler-Ross E. (1969). On death and dying. Macmillan.

[bibr26-0146167220934577] Langer E. J. Roth J. (1975). Heads I win, tails it’s chance: The illusion of control as a function of the sequence of outcomes in a purely chance task. Journal of Personality and Social Psychology, 32, 951–955.

[bibr27-0146167220934577] Lazarus R. S. (1966). Psychological stress and the coping process. McGraw-Hill.

[bibr28-0146167220934577] Loewenstein G. (1987). Anticipation and the valuation of delayed consumption. The Economic Journal, 97, 666–684.

[bibr29-0146167220934577] Maslow A. H. (1950). Self-actualizing people: A study of psychological health. Personality, Symposium, 1, 11–34.

[bibr30-0146167220934577] McGraw A. P. Mellers B. A. Ritov I. (2004). The affective costs of overconfidence. Journal of Behavioral Decision Making, 17, 281–295.

[bibr31-0146167220934577] Medvec V. H. Madey S. F. Gilovich T. (1995). When less is more: Counterfactual thinking and satisfaction among Olympic medalists. Journal of Personality and Social Psychology, 69, 603–610. 7473022 10.1037//0022-3514.69.4.603

[bibr32-0146167220934577] Mellers B. A. Schwartz A. Ho K. Ritov I. (1997). Decision affect theory: Emotional reactions to the outcomes of risky options. Psychological Science, 8, 423–429.

[bibr33-0146167220934577] Newby-Clark I. R. Ross M. (2003). Conceiving the past and future. Personality and Social Psychology Bulletin, 29, 807–818. 15018670 10.1177/0146167203029007001

[bibr34-0146167220934577] Pevalin D. J. (2000). Multiple applications of the GHQ-12 in a general population sample: An investigation of long-term retest effects. Social Psychiatry and Psychiatric Epidemiology, 35, 508–512. 11197926 10.1007/s001270050272

[bibr35-0146167220934577] Pyszczynski T. Holt K. Greenberg J. (1987). Depression, self-focused attention, and expectancies for positive and negative future life events for self and others. Journal of Personality and Social Psychology, 52, 994–1001. 3585706 10.1037//0022-3514.52.5.994

[bibr36-0146167220934577] Rogers C. R. (1961). On becoming a person: A therapist’s view of psychotherapy. Houghton-Mifflin.

[bibr37-0146167220934577] Rutledge R. B. Skandali N. Dayan P. Dolan R. J. (2014). A computational and neural model of momentary subjective well-being. Proceedings of the National Academy of Sciences, 111, 12252–12257. 10.1073/pnas.1407535111 PMC4143018 25092308

[bibr38-0146167220934577] Scheier M. E. Carver C. S. (1987). Dispositional optimism and physical well-being: The influence of generalized outcome expectancies on health. Journal of Personality, 55, 169–210. 3497256 10.1111/j.1467-6494.1987.tb00434.x

[bibr39-0146167220934577] Scheier M. F. Carver C. S. (1992). Effects of optimism on psychological and physical well-being: Theoretical overview and empirical update. Cognitive Therapy and Research, 16, 201–228.

[bibr40-0146167220934577] Scheier M. F. Carver C. S. (1993). On the power of positive thinking: The benefits of being optimistic. Current Directions in Psychological Science, 2, 26–30.

[bibr41-0146167220934577] Scheier M. F. Carver C. S. Bridges M. W. (1994). Distinguishing optimism from neuroticism (and trait anxiety, self-mastery, and self-esteem): A re-evaluation of the Life Orientation Test. Journal of Personality and Social Psychology, 67, 1063–1078. 7815302 10.1037//0022-3514.67.6.1063

[bibr42-0146167220934577] Seidlitz L. Diener E. (1993). Memory for positive versus negative life events: Theories for the differences between happy and unhappy persons. Journal of Personality and Social Psychology, 64, 654–664. 8473982 10.1037//0022-3514.64.4.654

[bibr43-0146167220934577] Sharot T. (2011). The optimism bias. Current Biology, 21, R941–R945. 22153158 10.1016/j.cub.2011.10.030

[bibr44-0146167220934577] Sharot T. Garrett N. (2016). Forming beliefs: Why valence matters. Trends in Cognitive Sciences, 20, 25–33. 26704856 10.1016/j.tics.2015.11.002

[bibr45-0146167220934577] Shepperd J. A. McNulty J. K. (2002). The affective consequences of expected and unexpected outcomes. Psychological Science, 13, 85–88. 11892785 10.1111/1467-9280.00416

[bibr46-0146167220934577] Sweeny K. Shepperd J. A. (2010). The costs of optimism and the benefits of pessimism. Emotion, 10, 750–753. 21038961 10.1037/a0019016

[bibr47-0146167220934577] Taylor S. E. Brown J. D. (1988). Illusion and well-being: A social psychological perspective on mental health. Psycho-logical Bulletin, 103, 193–210. 3283814

[bibr48-0146167220934577] Tenney E. R. Logg J. M. Moore D. A. (2015). (Too) optimistic about optimism: The belief that optimism improves performance. Journal of Personality and Social Psychology, 108, 377–399. 25751715 10.1037/pspa0000018

[bibr49-0146167220934577] Trivers R. (2000). The elements of a scientific theory of self-deception. Annals of the New York Academy of Sciences, 907, 114–131. 10818624 10.1111/j.1749-6632.2000.tb06619.x

[bibr50-0146167220934577] Weinstein N. D. (1980). Unrealistic optimism about future life events. Journal of Personality and Social Psychology, 39, 806–820.

[bibr51-0146167220934577] Weinstein N. D. Klein W. M. (1996). Unrealistic optimism: Present and future. Journal of Social and Clinical Psychology, 15, 1–8.

[bibr52-0146167220934577] Wilson A. E. Ross M. (2000). The frequency of temporal-self and social comparisons in people’s personal appraisals. Journal of Personality and Social Psychology, 78, 928–942. 10821199 10.1037//0022-3514.78.5.928

[bibr53-0146167220934577] Wilson A. E. Ross M. (2001). From chump to champ: People’s appraisals of their earlier and present selves. Journal of Personality and Social Psychology, 80, 572–584. 11316222 10.1037//0022-3514.80.4.572

[bibr54-0146167220934577] Young C. Holsteen K. (2017). Model uncertainty and robustness: A computational framework for multimodel analysis. Sociological Methods and Research, 46, 3–40.

