# Supplementary material for: Neither an Optimist Nor a Pessimist Be: Mistaken Expectations Lower Well-Being
Source: Pers Soc Psychol Bull. 2020 Jul 6;47(4):540–50. doi: 10.1177/0146167220934577 (PMC7961621; doi:10.1177/0146167220934577)
Supplement: PSPB_Methods_File – Supplemental material for Neither an Optimist Nor a Pessimist Be: Mistaken Expectations Lower Well-Being [file PSPB_Methods_File.docx]

**Neither an Optimist nor a Pessimist Be: Mistaken Expectations Lower Well-Being**

**METHODOLOGY FILE**

**Survey Instrument and Data Set**

The data set is the British Household Panel Survey (BHPS) 1991-2009 (Waves 1-18). The BHPS is a nationally representative longitudinal survey of more than 5,000 households (comprising approximately 12,000 individuals) which began in 1991, funded by the UK Economic and Social Research Council as a national and international multi-purpose research resource.

Questionnaires for all Waves of the BHPS can be located here:

<https://www.iser.essex.ac.uk/bhps/documentation/pdf_versions/survey_docs/>

The questionnaires for Wave 15 of the BHPS can be located here:

<https://www.iser.essex.ac.uk/bhps/documentation/pdf_versions/questionnaires/bhpsw15q.pdf>

The sample used for analysis is restricted to the original BHPS sample covering Great Britain (The original sample excludes Northern Ireland. Booster samples for Wales and Scotland recruited in 1999 and a sample for Northern Ireland recruited in 2001 are also excluded) and to individuals who were observed in all 18 waves and gave valid responses to the dependent and independent variables used in the subsequent analysis.

Estimation procedures are described in detail in the article. Below detailed variable codebook descriptions

**Variable Definitions**

1. **General Health Questionnaire**

Here are some questions regarding the way you have been feeling over the last few weeks. For each question please tick the box next to the answer that best describes the way you have felt.

Have you recently....

a) been able to concentrate on whatever you're doing?

Better than usual ........................

Same as usual .............................

Less that usual............................

Much less than usual..................

b) lost much sleep over worry?

Not at all.....................................

No more than usual.....................

Rather more than usual...............

Much more than usual................

c) felt that you were playing a useful part in things?

More than usual..........................

Same as usual .............................

Less so than usual.......................

Much less than usual..................

d) felt capable of making decisions about things?

More so than usual .....................

Same as usual .............................

Less so than usual.......................

Much less capable ......................

e) felt constantly under strain?

Not at all.....................................

No more than usual.....................

Rather more than usual...............

Much more than usual................

f) felt you couldn't overcome your difficulties?

Not at all.....................................

No more than usual.....................

Rather more than usual...............

Much more than usual................

g) been able to enjoy your normal day-to-day activities?

More so than usual .....................

Same as usual .............................

Less so than usual.......................

Much less than usual..................

h) been able to face up to problems?

More so than usual .....................

Same as usual .............................

Less able than usual....................

Much less able............................

i) been feeling unhappy or depressed?

Not at all.....................................

No more than usual.....................

Rather more than usual...............

Much more than usual................

j) been losing confidence in yourself?

Not at all.....................................

Not more than usual ...................

Rather more than usual...............

Much more than usual................

k) been thinking of yourself as a worthless person?

Not at all.....................................

No more than usual.....................

Rather more than usual...............

Much more than usual................

l) been feeling reasonably happy, all things considered?

More so than usual .....................

About the same as usual.............

Less so than usual.......................

Much less than usual..................

These 12 questions are named in the BHPS as follows: **GHQA-GHQL**

1. **Life Satisfaction**

Here are some questions about how you feel about your life. Please tick the number which you feel best describes how dissatisfied or satisfied you are with the following aspects of your current situation.

1 = NOT SATISFIED AT ALL 7 = COMPLETELY SATISFIED

Using the same scale how dissatisfied or satisfied are you with your life overall?

Respondents were asked this question in waves 6–10 and 12–18 of the BHPS.

This variable is named in the BHPS as follows: **LFSATO**

1. **Financial Expectations**

Looking ahead, how do you think you will be financially a year from now, will you be. . . .

Better off.......................................... 1

Worse off than you are now........... 2

Or about the same?......................... 3

Don't know....................................... 8

This variable is named in the BHPS as follows: **FISITX**

1. **Financial Realizations**

Would you say that you yourself are better off or worse off financially than you were a year ago?

Better off....................................... 1

Worse off....................................... 2

About the same............................. 3

Don't know.................................... 8

This variable is named in the BHPS as follows: **FISITC**

1. **Control Variables**

Control variables are: gender, age (in linear form); marital status; the number of dependent children in the household; economic activity; educational attainment; housing tenure; logged monthly household income (deflated) and number of cigarettes smoked.

1. **Age and Gender**

Please write in your date of birth:

and tick male or female

These variables are named in the BHPS as follows: **DOBY** and **SEX**

1. **Marital Status**

‘Are you/is .... currently married, living with a partner, widowed, divorced or separated or have you/they never been married?’

Married...............1

Living as couple..2

Widowed.............3

Divorced .............4

Separated.............5

Never married.....6

Under 16 ............0

This variable is named in the BHPS as follows: **MASTAT**

1. **Number of Dependent Children in Household**

This is a derived variable.

Dependent Children unlike the standard definition of a child for fieldwork purposes (i.e. under 16), a dependent child has been defined for use in derived variable construction as one aged under 16, or aged 16-18 and in school or non-advanced further education, not married and living with parent.

This measures the number of dependent children in the household aged 16 and over. Dependent children are defined as those unmarried, aged under 19, and in school or non-advanced further education. This variable is missing if any person in the household has a missing age, or if a child in the age range has a missing employment status.

This variable is named in the BHPS as follows: **NCH1618**

1. **Economic Activity**

Please look at this card and tell me which best describes her/his current situation?

Self employed..................................................... 01

In paid employment (full or part-time)................ 02 Unemployed....................................................... 03

Retired from paid work altogether...................... 04

On maternity leave............................................. 05

Looking after family or home.............................. 06

Full-time student/at school............................... 07

Long term sick or disabled................................. 08

On a government training scheme...................... 09

Something else (PLEASE GIVE DETAILS) …. 10

Don't know ........................................................ 98

This variable is named in the BHPS as follows: **JBSTAT**

1. **Educational Attainment**

This is a derived variable. The definition of categories are as follows, with respondents allocated to the highest category into which they fall, or into category 7 if no academic qualifications:

1. Higher Degree is held

2. 1st Degree

3. Higher National Certificate/Diploma or teaching

4. A Levels, Scottish Higher Grades, Scottish School Leaving Certificate Higher Grade, Scottish Certificate of Sixth Year Studies, Higher School Certificate, Ordinary National Certificate/Diploma, BEC/TEC/BTEC National/General Certificate or Diploma or City & Guilds Certificate (Advanced/Final/Part II).

5. O Levels (pre 1975), O Level grades A-c (1975 or later), GCSE grades A-C, CSE grade 1, Scottish O Grades (pass or bands A-C or 1-3), Scottish School Leaving Certificate Lower Grade, School Certificate or Matric, Scottish Standard Grade Level 1-3 or City & Guilds Certificate (Craft/Intermediate/Ordinary/Part I)

6. CSE Grades 2-5, O Level grades D-E, GCSE grades D-G, Scottish SCE Ordinary Grade bands D-E or 4-5 or Scottish Standard Grade levels 4-7

The data in this variable is up-dated each year to include the most recent qualifications of new entrants and existing panel members. The variable shows the current status of the respondent and there is no need for the user to add the recently acquired qualifications to the first, or any subsequent, iteration of this variable.

This variable is named in the BHPS as follows: **QFACHI**

1. **Housing Tenure**

This question is a derived variable and uses MGHAVE RENTLL RENTF HSOWND

**NMGHAVE:**

Is this accommodation:

Owned outright ............................. 1

Or is it being bought with a mortgage or a loan?............. 2

**NRENTLL:**

Who is the accommodation rented from or provided by?

Local Authority/Council/ Northern Ireland Housing Executive.................... 01

New Town Commission or Corporation.......................... 02

Property company ......................................................... 03

Scottish Homes/Communities Scotland (Scottish Special Housing Association) ................ 04

Other Housing association, cooperative or charitable trust ................................................... 05 Employer....................................................................... 06

Other organisation (SPECIFY) ......................................................................07

**NRENTF:**

Do you rent your accommodation. . .

Furnished ................1

Partly furnished .......2

Or unfurnished? ......3

**HSOWND:**

Does your household own or rent this accommodation or does it come rent-free?

Owned/being bought on mortgage ....................... 1

Shared ownership (part-owned part-rented) ......... 2

Rented ................................................................. 3

Rent free ............................................................. 4

Other (SPECIFY) ................................................... 5

This variable is named in the BHPS as follows: **TENURE**

1. **Monthly Household Income**

This is a derived variable.

This variable sums the values of total income in the month before interview for individuals in the household (using wFIMN).

**FIMN:**

This variable is the sum of non-labour income, and labour income. Income from second jobs is also added if non- missing. For proxy cases estimated total personal income is used, taking the midpoint of each band (£3300 pcm for the highest band). Includes imputed data.

This variable is named in the BHPS as follows: **FIHHMN**

1. **Number of Cigarettes Smoked**

Approximately how many cigarettes a day do you usually smoke, including those you roll yourself?

IF VARIES, PROMPT FOR DAILY AVERAGE OVER LAST WEEK NUMBER: PER DAY

Number:

Less than 1 = 00

This variable is named in the BHPS as follows: **ONCIGS**

1. **Short 15-item Big-Five inventory (BFI-15)**

I see myself as someone who . . .

1 = DOES NOT APPLY TO ME AT ALL 7 = APPLIES TO ME PERFECTLY

a) Is sometimes rude to others OPTRT5A1*

b) Does a thorough job OPTRT5C1

c) Is talkative OPTRT5E1

d) Worries a lot OPTRT5N1

e) Is original, comes up with new ideas OPTRT5O1

f) Has a forgiving nature OPTRT5A2

g) Tends to be lazy OPTRT5C2*

h) Is outgoing, sociable OPTRT5E2

i) Gets nervously easily OPTRT5N2

j) Values artistic, aesthetic experiences OPTRT5O2

k) Is considerate and kind to almost everyone OPTRT5A3

l) Does things efficiently OPTRT5C3

m) Is reserved OPTRT5E3*

n) Is relaxed, handles stress well OPTRT5N3*

o) Has an active imagination OPTRT5O3

Conscientiousness (OPTRT5C1, OPTRT5C2, OPTRT5C3)

Extraversion (OPTRT5E1, OPTRT5E2, OPTRT5E3)

Agreeableness (OPTRT5A1, OPTRT5A2, OPTRT5A3)

Openness (OPTRT5O1, OPTRT5O2, OPTRT5O3)

Neuroticism (OPTRT5N1, OPTRT5N2, OPTRT5N3)

*Items reversed.
