# Supplementary material for: Neither an Optimist Nor a Pessimist Be: Mistaken Expectations Lower Well-Being
Source: Pers Soc Psychol Bull. 2020 Jul 6;47(4):540–50. doi: 10.1177/0146167220934577 (PMC7961621; doi:10.1177/0146167220934577)
Supplement: Supplemental_Materials_v1.1 – Supplemental material for Neither an Optimist Nor a Pessimist Be: Mistaken Expectations Lower Well-Being [file Supplemental_Materials_v1.1.docx]

**Supplemental Materials**

**Table S1.** Descriptive Statistics

| Variable | Mean | Standard Deviation | Minimum | Maximum |
| --- | --- | --- | --- | --- |
| GHQ | 10.90 | 3.12 | 3.17 | 26.83 |
| Life Satisfaction | 5.33 | 0.84 | 1.17 | 7.00 |
| Financial Expectations | 0.13 | 0.31 | -0.94 | 1.00 |
| Financial Realizations | 0.01 | 0.31 | -1.00 | 0.94 |
| Financial Forecast Error | 0.12 | 0.28 | -0.71 | 1.41 |
| Female | 0.55 |  | 0 | 1 |
| Age (years) | 47.64 | 13.84 | 23.50 | 88.50 |
| Employee | 0.60 |  | 0.00 | 1.00 |
| Self-Employed | 0.07 |  | 0.00 | 1.00 |
| Unemployed | 0.02 |  | 0.00 | 0.72 |
| Full-time student | 0.07 |  | 0.00 | 0.94 |
| Retired | 0.19 |  | 0.00 | 1.00 |
| Economically inactive | 0.04 |  | 0.00 | 1.00 |
| Single, never married | 0.10 |  | 0.00 | 1.00 |
| Widowed/divorced/separated | 0.11 |  | 0.00 | 1.00 |
| Married - partner employed | 0.56 |  | 0.00 | 1.00 |
| Married - partner not employed | 0.24 |  | 0.00 | 1.00 |
| Number of dependent children in the household | 0.61 | 0.74 | 0.00 | 4.50 |
| University or college degree | 0.13 |  | 0.00 | 1.00 |
| HND/HNC | 0.08 |  | 0.00 | 1.00 |
| A-levels | 0.19 |  | 0.00 | 1.00 |
| O-levels/GCSE’s | 0.32 |  | 0.00 | 1.00 |
| No formal qualification | 0.27 |  | 0.00 | 1.00 |
| Own house outright | 0.30 |  | 0.00 | 1.00 |
| Own house with mortgage | 0.55 |  | 0.00 | 1.00 |
| Rents house, private sector | 0.05 |  | 0.00 | 1.00 |
| Rents house, social sector | 0.10 |  | 0.00 | 1.00 |
| Log monthly household income (deflated) | 7.74 | 0.54 | 5.88 | 9.33 |
| Number of cigarettes smoked | 2.91 | 6.36 | 0.00 | 64.44 |
| $N$ | 1,601 |  |  |  |

Note: Summary statistics of individual time-averaged dependent and independent variables. Educational attainment is measured through a series of dummy variables indicating the highest level of attainment. These are: university or college degree level at undergraduate or postgraduate level; HND (Higher National Diplomas) and HNC (Higher National Certificates) which are work-related, or vocational, higher education qualifications; A-levels or equivalent (post-compulsory examinations taken at 18 as qualifying exams for college or university entrance); GCSE or O-levels (age 16 schooling attainment qualifications); and no formal qualifications.

**Table S2.** Tests of Knife-Edge Specification. Second-Degree Polynomial Ordinary Least Squares (OLS) Regressions Measuring the Impact of Expectations and Realizations on Psychological Well-Being

|  | Dependent variable: GHQ | | | |  | Dependent variable: Life Satisfaction | | | |  |
| --- | --- | --- | --- | --- | --- | --- | --- | --- | --- | --- |
|  | Regression 1 | | | |  | Regression 2 | | | |  |
|  |  | 95% CI | |  |  |  | 95% CI | |  |  |
| Predictors | $b$ | Low | High | $t$ | Stability | $b$ | Low | High | $t$ | Stability |
| $\bar{E}_{i}$ | -1.39 | -2.32 | -0.45 | -2.91** | [100, 100] | 0.20 | -0.06 | 0.47 | 1.50 | [99, 21] |
| $\bar{E}_{i}^{2}$ | 2.69 | 1.08 | 4.30 | 3.27** | [100, 100] | -1.01 | -1.45 | -0.57 | -4.50** | [100, 100] |
| $\bar{R}_{i}$ | -0.94 | -1.72 | -0.16 | -2.36* | [100, 98] | 0.44 | 0.23 | 0.64 | 4.22** | [100, 100] |
| $\bar{R}_{i}^{2}$ | 1.61 | 0.04 | 3.18 | 2.02* | [100, 68] | -0.47 | -0.91 | -0.04 | -2.14* | [100, 82] |
| $\bar{E}_{i}\times\bar{R}_{i}$ | -4.49 | -6.70 | -2.28 | -3.98** | [100, 100] | 1.01 | 0.38 | 1.63 | 3.15** | [100, 100] |
| $N$ | 1,601 |  |  |  |  | 1,601 |  |  |  |  |

Note: Control variables are gender and a series of individual time averaged control variables for age (in linear form), marital status, the number of dependent children in the household, economic activity, educational attainment, housing tenure, logged monthly household income (deflated), number of cigarettes smoked and region of residence. Following Young and Holsteen (2017), coefficients represent the mean coefficient estimate across all combinations of possible control variables. The procedure yields 1,024 unique combinations of control variables. Standard errors used in the calculations are the combined total of the average sampling and modelling standard error. Stability = sign stability (%) and statistical significance rate (%) for our coefficient estimates across the 1,024 unique combinations of control variables, with $p< .05$ being the threshold for statistical significance. For example, [100, 100] would indicate that with this list of possible control variables, it is not possible to find an opposite signed or even nonsignificant estimate. CI = confidence interval.

$$*p< .05. **p< .01.$$

**Table S3.** Tests of Knife-Edge Specification. Ordinary Least Squares (OLS) Regressions Measuring the Impact of Forecasting Errors on Psychological Well-Being

|  | Dependent variable: GHQ | | | |  | Dependent variable: Life Satisfaction | | | |  |
| --- | --- | --- | --- | --- | --- | --- | --- | --- | --- | --- |
|  | Regression 1 | | | |  | Regression 2 | | | |  |
|  |  | 95% CI | |  |  |  | 95% CI | |  |  |
| Predictors | $b$ | Low | High | $t$ | Stability | $b$ | Low | High | $t$ | Stability |
| $\bar{FE}_{i}$ | -1.21 | -2.02 | -0.40 | -2.93** | [100, 100] | 0.07 | -0.16 | 0.30 | 0.60 | [88, 0] |
| $\bar{FE}_{i}^{2}$ | 2.22 | 1.14 | 3.30 | 4.02** | [100, 100] | -0.61 | -0.92 | -0.30 | -3.84** | [100, 100] |
| $\bar{R}_{i}$ | -2.18 | -2.85 | -1.51 | -6.34** | [100, 100] | 0.52 | 0.30 | 0.73 | 4.70** | [100, 100] |
| $N$ | 1,601 |  |  |  |  | 1,601 |  |  |  |  |

Note: Control variables are gender and a series of individual time averaged control variables for age (in linear form), marital status, the number of dependent children in the household, economic activity, educational attainment, housing tenure, logged monthly household income (deflated), number of cigarettes smoked and region of residence. Following Young and Holsteen (2017), coefficients represent the mean coefficient estimate across all combinations of possible control variables. The procedure yields 1,024 unique combinations of control variables. Standard errors used in the calculations are the combined total of the average sampling and modelling standard error. Stability = sign stability (%) and statistical significance rate (%) for our coefficient estimates across the 1,024 unique combinations of control variables, with $p< .05$ being the threshold for statistical significance. For example, [100, 100] would indicate that with this list of possible control variables, it is not possible to find an opposite signed or even nonsignificant estimate. CI = confidence interval.

$$*p< .05. **p< .01.$$

**Table S4.** Controlling for the Big-Five. Second-Degree Polynomial Ordinary Least Squares (OLS) Regressions Measuring the Impact of Expectations and Realizations on Psychological Well-Being

|  | Dependent variable: GHQ | | | | Dependent variable: Life Satisfaction | | | |
| --- | --- | --- | --- | --- | --- | --- | --- | --- |
|  | Regression 1 | | | | Regression 2 | | | |
|  |  | 95% CI | |  |  | 95% CI | |  |
| Predictors | $b$ | Low | High | $t$(*df* = 1559) | $b$ | Low | High | $t$(*df* = 1559) |
| $\bar{E}_{i}$ | -0.83 | -1.58 | -0.07 | -2.15* | 0.12 | -0.10 | 0.33 | 1.08 |
| $\bar{E}_{i}^{2}$ | 2.23 | 0.88 | 3.57 | 3.25** | -0.87 | -1.24 | -0.49 | -4.51** |
| $\bar{R}_{i}$ | -0.99 | -1.63 | -0.36 | -3.06** | 0.46 | 0.28 | 0.63 | 5.02** |
| $\bar{R}_{i}^{2}$ | 1.20 | -0.16 | 2.56 | 1.73 | -0.38 | -0.76 | 0.00 | -1.94 |
| $\bar{E}_{i}\times\bar{R}_{i}$ | -2.94 | -4.92 | -0.95 | -2.90** | 0.65 | 0.10 | 1.21 | 2.31* |
| Conscientiousness | -0.20 | -0.33 | -0.06 | -2.91** | 0.10 | 0.06 | 0.13 | 5.02** |
| Extraversion | -0.05 | -0.17 | 0.07 | -0.85 | 0.06 | 0.03 | 0.09 | 3.53** |
| Agreeableness | 0.04 | -0.10 | 0.18 | 0.57 | 0.06 | 0.02 | 0.10 | 2.88** |
| Openness | 0.04 | -0.08 | 0.17 | 0.69 | -0.01 | -0.05 | 0.02 | -0.77 |
| Neuroticism | 1.16 | 1.05 | 1.26 | 21.08** | -0.20 | -0.23 | -0.17 | -12.90** |
| *F*-test | 4.09** |  |  |  | 9.86** |  |  |  |
| R-squared | 0.378 |  |  |  | 0.330 |  |  |  |
| $N$ | 1,600 |  |  |  | 1,600 |  |  |  |

Note: Personality traits - conscientiousness, extraversion, agreeableness, openness, neuroticism – are measured using the short 15-item Big-Five inventory (BFI-15). Each trait is based on a level of agreement with three statements, assessed on a seven-point scale. Response are added across each set of the three statements and then divided by the number of items over which the sum is calculated. All regressions include a control variable for gender and a series of individual time-averaged control variables for age (in linear form), marital status, the number of dependent children in the household, economic activity, educational attainment, housing tenure, logged monthly household income (deflated), number of cigarettes smoked and region of residence. Full results are available on request. CI = confidence interval. *F*-test is for the three second-degree polynomial terms.

$$*p< .05. **p< .01.$$

**Table S5.** Controlling for the Big-Five. Ordinary Least Squares (OLS) Regressions Measuring the Impact of Forecasting Errors on Psychological Well-Being

|  | Dependent variable: GHQ | | | | Dependent variable: Life Satisfaction | | | |
| --- | --- | --- | --- | --- | --- | --- | --- | --- |
|  | Regression 1 | | | | Regression 2 | | | |
|  |  | 95% CI | |  |  | 95% CI | |  |
| Predictors | $b$ | Low | High | $t$(*df* = 1561) | $b$ | Low | High | $t$(*df* = 1561) |
| $\bar{FE}_{i}$ | -0.61 | -1.30 | 0.07 | -1.76 | -0.01 | -0.20 | 0.18 | -0.12 |
| $\bar{FE}_{i}^{2}$ | 1.58 | 0.61 | 2.55 | 3.19** | -0.45 | -0.73 | -0.18 | -3.25** |
| $\bar{R}_{i}$ | -1.63 | -2.16 | -1.10 | -6.01** | 0.45 | 0.30 | 0.60 | 5.95** |
| Conscientiousness | -0.20 | -0.34 | -0.07 | -2.93** | 0.10 | 0.06 | 0.14 | 5.04** |
| Extraversion | -0.05 | -0.17 | 0.07 | -0.86 | 0.06 | 0.03 | 0.09 | 3.52** |
| Agreeableness | 0.04 | -0.10 | 0.18 | 0.53 | 0.06 | 0.02 | 0.10 | 2.99** |
| Openness | 0.05 | -0.07 | 0.17 | 0.80 | -0.02 | -0.05 | 0.02 | -1.07 |
| Neuroticism | 1.15 | 1.05 | 1.26 | 21.05** | -0.20 | -0.23 | -0.17 | -12.70** |
| R-squared | 0.377 |  |  |  | 0.322 |  |  |  |
| $N$ | 1,600 |  |  |  | 1,600 |  |  |  |

Note: Personality traits - conscientiousness, extraversion, agreeableness, openness, neuroticism – are measured using the short 15-item Big-Five inventory (BFI-15). Each trait is based on a level of agreement with three statements, assessed on a seven-point scale. Response are added across each set of the three statements and then divided by the number of items over which the sum is calculated. All regressions include a control variable for gender and a series of individual time-averaged control variables for age (in linear form), marital status, the number of dependent children in the household, economic activity, educational attainment, housing tenure, logged monthly household income (deflated), number of cigarettes smoked and region of residence. Full results are available on request. CI = confidence interval.

$$*p< .05. **p< .01.$$


**Figure S1.** Scatterplot of expectations and realizations. The 45-degree line represents the equality of expectations and realizations.

**Figure S2.** Histogram of GHQ and life satisfaction.

**Figure S3.** Controlling for the Big-Five. The quadratic relationship between expectations, forecast errors and psychological well-being. We also include as bar charts the slope estimates (derivative) of the quadratic relationships, with the 95% confidence intervals included. Top left (Regression 1, Table S4): the turning point of the quadratic prediction is 0.19, with a 95% confidence interval, or CI = [0.06, 0.31]. Top right (Regression 2, Table S4): the turning point of the quadratic prediction is 0.07, with a 95% CI = [-0.04, 0.17]. Bottom left (Regression 1, Table S5): the turning point of the quadratic prediction is 0.19, with a 95% CI = [0.02, 0.37]. Bottom right (Regression 2, Table S5): the turning point of the quadratic prediction is -0.01, with a 95% CI = [-0.23, 0.21]. All of these turning points and the respective 95% confidence intervals lie comfortably within the range of observable values in the data.

**Does well-being determine forecast accuracy?**

The “precision hypothesis” is that fewer stresses enable clearer, more accurate forecasts. In that case it is well-being that determines optimism and not the reverse.

To investigate whether this effect is present, we performed a within-person analysis of whether changes to an individuals’ psychological well-being influenced the accuracy of their financial expectations.

**Method**

**Participants**

The sample used for the subsequent analysis is restricted to the original BHPS sample covering Great Britain and to individuals who were observed in all 18 waves and gave valid responses to the dependent and independent variables used in the subsequent analysis. This yields a balanced panel of 1,601 individuals.

**Analytic Strategy**

Using within-person variation we formally estimate whether changes to an individuals’ psychological well-being influenced the accuracy of financial expectations. We used a correlated random-effects ordered probit model, following Mundlak (1978) and Wooldridge (2005). Here, the dependent variable is our ordinal financial realization variable ${(R}_{it+1})$, measured for individual, $i$, at $time t+1$. The predictor variables include our ordinal financial expectations variable measured at $time t$ $(E_{it})$, psychological well-being at $time t$ ${(W}_{it}^{j})$ for well-being measure $j=(GHQ, LS)$ and the interaction of these two predictor variables. We input GHQ into the equation in linear form whereas life satisfaction is treated as an ordinal variable. Specifically, we estimated the following fixed-effects equation via an ordered probit correlated random-effects approach (to simplify, we omitted all control variables from the notation):

$$R_{it+1}=b_{0}+b_{1}E_{it}+b_{2}W_{it}^{j}+b_{3}\left( E_{it}\times W_{it}^{j} \right)+{\alpha_{i}+e}_{it}$$

As usual, $\alpha_{i}$ is an unobservable individual-specific effect and $e_{\mathrm{it}}$is the idiosyncratic error term which picks up the effect of time-varying unobservable determinants. In the equation, we control for a range of individual time-varying socioeconomic and sociodemographic controls. These control variables are: age (in linear form); marital status; the number of dependent children in the household; economic activity; educational attainment; housing tenure; logged monthly household income (deflated); number of cigarettes smoked and region of residence.

**Results**

The key results of this equation are summarized in Figure S4 were we plot the predicted probabilities of each financial realization at $time t+1$, for every combination of financial expectation and psychological well-being status at$time t$. If an individual’s financial expectations are more random when their psychological well-being is low, the forecast will be less likely to be correct. In fact, changes to psychological well-being have a negligible influence on the probability of forecast accuracy as is illustrated in Figure S4. Figure S4 also clearly illustrates that financial expectations are in part rational as the probability of a given realization at $time t+1$ is highest for the matching expectation at$time t$. However, changes to individual well-being do not increase or decrease the likelihood that expectations will be fulfilled.

**Figure S4.** The predicted probability of financial realizations at $time t+1$, for every combination of financial expectation and psychological well-being status at$time t$. Error bars illustrate the 95% confidence intervals of the predictions.

**References**

Mundlak, Y. (1978). On the pooling of time series and cross section data. *Econometrica: Journal of the Econometric Society*, *46*, 69-85.

Wooldridge, J. M. (2005). Simple solutions to the initial conditions problem in dynamic, nonlinear panel data models with unobserved heterogeneity. *Journal of Applied Econometrics*, *20*, 39-54.
